# Supplementary material for: Integrated people-centered eye care: A scoping review on engaging communities in eye care in low- and middle-income settings
Source: PLoS One. 2023 Jan 19;18(1):e0278969. doi: 10.1371/journal.pone.0278969 (PMC9851534; doi:10.1371/journal.pone.0278969)
Supplement: S1 Table — (DOCX) [file pone.0278969.s002.docx]

| **SEARCH GROUP #1 (Eye care terms)** |
| --- |
| 1 exp Eye Diseases/  2 exp Ophthalmology/  3 optometry/  4 ((eye$ or ocular or vision) adj2 (care or health or service$)).tw,kf.  5 (visual adj1 acuity).tw,kf.  6 (ophthalm$ or optomet$).tw,kf.  7 retinopath$.tw,kf.  8 (glaucoma$ or ocular hypertension).tw,kf.  9 cataract$.tw,kf.  10 (dilated adj2 fundus).tw,kf.  11 (retinal adj2 exam$).tw,kf.  12 (refractive adj1 error$).tw,kf.  13 (spectacle$ or glasses or eyeglasses or eye glasses).tw,kf.  14 blindness.tw,kf.  15 ((vision or visual$) adj1 impair$).tw,kf.  16 (trachoma$ or trichia$).tw,kf.  17 (myopic degeneration or macular degeneration or age-related macular degeneration or armd).tw,kf.  18 (amblyopi$ or strabism$).tw,kf.  19 (conjunctiv$ or pteryg$).tw,kf.  20 (myop$ or hyperop$ or hypermetrop$ or anisometrop$ or ammetrop$ or astigmati$ or presbyop$).tw,kf.  21 (ptosis or entropion or ectropion or blepharitis or chalazion or hordeolum).tw,kf.  22 (cornea$ adj2 (ulcer or infection)).tw,kf.  23 (keratitis or keratoconus).tw,kf.  24 (dry eye disease or ocular surface disease).tw,kf.  25 ((low adj1 vision) or vision rehabilitation or visual rehabilitation).tw,kf.  26 (onchocerciasis or measles or rubella or vitamin a deficiency).tw,kf.  27 ((diabet$ or proliferat$) adj3 retinopath$).tw,kf.  28 (diabet$ adj3 (eye$ or vision or visual$ or sight$)).tw,kf.  29 red eye.tw,kf.  30 eye problem.tw,kf.  31 eye health.tw,kf.  32 (ocular adj1 (injur$ or trauma)).tw,kf.  33 or/1-32 |
| **SEARCH GROUP #2 (Community engagement terms)** |
| 34 community based.tw,kf.  35 local community.tw,kf.  36 community volunteer$.tw,kf.  37 key informant$.tw,kf.  38 local school$.tw,kf.  39 school-based.tw,kf.  40 community partnership$.tw,kf.  41 accountability.tw,kf.  42 outreach.tw,kf.  43 community participation.tw,kf.  44 community engagement.tw,kf.  45 community advisory.tw,kf.  46 community consultation$.tw,kf.  47 community-based participatory research.tw,kf.  48 community-based research.tw,kf.  49 (community counselling or community counseling).tw,kf.  50 community health planning.tw,kf.  51 community advocacy.tw,kf.  52 community health service$.tw,kf.  53 community action.tw,kf.  54 citizen participation.tw,kf.  55 public participation.tw,kf.  56 citizen engagement.tw,kf.  57 community network$.tw,kf.  58 exp community-institutional relations/  59 or/34-58 |
| **SEARCH GROUP #3 (Intervention terms)** |
| 60 intervention$.tw,kf.  61 program$.tw,kf.  62 programme$.tw,kf.  63 initiative$.tw,kf.  64 incentive$.tw,kf.  65 campaign$.tw,kf.  66 strategy.tw,kf.  67 strategies.tw,kf.  68 health education.tw,kf.  69 or/60-69 |
| **SEARCH GROUP #4** |
| 70 33 and 59 and 69  71 limit 55 to yr="2011 -Current" |
