# Supplementary material for: Integrated people-centered eye care: A scoping review on engaging communities in eye care in low- and middle-income settings
Source: PLoS One. 2023 Jan 19;18(1):e0278969. doi: 10.1371/journal.pone.0278969 (PMC9851534; doi:10.1371/journal.pone.0278969)
Supplement: S2 Table — *Multiple = At least 3 funding sources associated with supporting the study are acknowledged; ^Epub ahead of print; CDD = community drug distributor; CDTI = community-directed treatment intervention; CHW = community health worker; FGD = focus group discussion. (DOCX) [file pone.0278969.s003.docx]

| **First Author (Reference)** | **Year** | **Country** | **Participant type**  **Sample size (n)** | **Methodology** | **Study quality** | **Funding source*** |
| --- | --- | --- | --- | --- | --- | --- |
| Adekeye [60] | 2020 | Nigeria | Key informants (n=42), frontline health facility staff (n=90), CDDs (n=84), teachers (n=89) | Qualitative | 0.55 | COUNTDOWN research consortium |
| Adhisesha Reddy [73] | 2018 | Multiple | Hospital staff (n=35), School staff (n=13), community personnel (n=21) | Mixed-methods | 0.43 | USAID |
| Agyemang [86] | 2018 | Ghana | Adults (2002: n=614; 2006: n=692; 2013: n=447), CDDs (n=28) | Observational | 0.75 | Bernhard Nocht Institute for Tropical Medicine |
| Asegedew [26] | 2019 | Ethiopia | Community volunteers (n=675) | Observational | 0.67 | Not stated |
| Bechange [84] | 2021 | Pakistan | Teachers (n=14), optometrists (n=3) | Qualitative | 0.80 | USAID |
| Bhondve [36] | 2019 | India | Adults (n=358) | Mixed-methods | 0.66 | Nil |
| Bof [33] | 2019 | Democratic Republic of Congo | Villages (n=42,778) | Observational | 0.95 | Not stated |
| Bof [72] | 2019 | Democratic Republic of Congo | Community members and key informants (n=106) | Qualitative | 0.80 | Nil |
| Bof [89] | 2018 | Democratic Republic of Congo | Patients (n=945) | Observational | 0.86 | Nil |
| Chan [17] | 2021 | Tanzania | Children (n=11134) | Interventional | 0.68 | USAID |
| Chariwala [51] | 2020 | India | Adults (n=300) | Interventional | 0.75 | The Queen Elizabeth Diamond Jubilee Trust |
| Chen [18] | 2021 | Tanzania | Health teachers (n=68), Regular teachers (n=81), Children (n=3084) | Observational | 0.82 | Wilmer Eye Institute |
| Chimpololo [27] | 2019 | Malawi | NGHOs (n=11) | Observational | 0.58 | Not stated |
| Chingono [35] | 2021 | Zimbabwe | Key informants (n=25), parents (n=95), adolescents (n=96) | Qualitative | 0.80 | Multiple |
| Demissie [28] | 2020 | Ethiopia | Infants (2013: n=5720; 2014: n=5774; 2015: n=5002; 2016: n=5289) | Mixed-methods | 0.95 | International Initiative for Impact Evaluation and Pfizer Foundation |
| Dissak-Delon [66] | 2019 | Cameroon | District officials (n=3), Chiefs of Health Area (n=6), Resigned CDD (n=7), health area committee (n=18), CDD (n=35) | Qualitative | 0.80 | Belgian Academie de Recherche et d'Enseignement Superieur |
| Dissak-Delon [61] | 2019 | Cameroon | Canton leader (n=1), Community leaders (n=6), CDTI adverse (n=4), elders (n=36), youths (n=41) | Qualitative | 0.90 | Belgian Academie de Recherche et d'Enseignement Superieur |
| Duamor [37] | 2017 | Cameroon | Chiefs of Bureau Health (n=5), Chiefs of Centres (n=12), CDDs (n=40), Community Heads (n=24) | Mixed-methods | 0.58 | Nil |
| Duke [39] | 2013 | Nigeria | Key informants (n=742) | Qualitative | 0.65 | Pediatric Cataract Initiative of the Bausch+Lomb and Lions Club International Foundation |
| Dursun [82] | 2015 | Turkey | Students (n=40) | Interventional | 0.85 | Not stated |
| Endale [67] | 2015 | Ethiopia | Adults (n=308) | Observational | 0.91 | Addis Ababa University |
| Ganesh [74] | 2018 | India | Children (n=345) | Observational | 0.90 | NEI and USAID |
| Gatobu [69] | 2017 | Ethiopia | Key informants (n=14), Adults (n>120) | Mixed-methods | 0.58 | Global Affairs Canada |
| Gelaye [48] | 2014 | Ethiopia | Students (n=669) | Interventional | 0.82 | Multiple |
| Gil Cuesta [40] | 2020 | Guinea | Key informants (n=14), Caregivers (n=68) | Qualitative | 0.70 | Nil |
| Greene [44] | 2015 | Tanzania | Individuals (n=27473) | Interventional | 0.81 | LCIF |
| Guo [21] | 2019 | China | Children (n=373) | Interventional | 0.69 | National Natural Science Foundation of China |
| Gupta [29] | 2018 | Tanzania | Individuals (n=28), CHWs (n=unknown) | Qualitative | 0.45 | Unassigned |
| Hobday [19] | 2015 | Timor-Leste | Students (n=384) | Mixed-methods | 0.70 | Nil |
| Jacob [79] | 2018 | Uganda | Villages (n=12, not individuals) | Interventional | 0.73 | National Institute of Allergy and Infectious Diseases |
| Jaiswal [38] | 2016 | India | Individuals (n=272) | Mixed-methods | 0.67 | Multiple |
| Jin [22] | 2015 | China | Students (n=3051) | Interventional | 0.58 | Research Special Fund for Public Welfare Industry of Health |
| Kalua [75] | 2012 | Malawi | Health surveillance assistants (n=59), Key informants (n=64), Children (n=167) | Mixed-methods | 0.54 | BCPB |
| Kamadjeu [34] | 2015 | Somalia | Children vaccinated (n=23099), Children (vit A supp)(n=12556) | Observational | 0.11 | Global Polio Eradication Initiative |
| Kamga [68] | 2018 | Cameroon | Community leades (n=69), Interviewees (n=2942), CDDs (n=83) | Observational | 0.82 | Belgian University Cooperation |
| Karan [59] | 2014 | India | Patients (n=97) | Interventional | 0.61 | Nil |
| Karimurio [78] | 2017 | Kenya | Individuals (n=22912) | Observational | 0.65 | Operation Eyesight Universal |
| Kassa [65] | 2020 | Ethiopia | Mothers/caregivers (n=813) | Observational | 0.95 | Hawassa University |
| Katabarwa [62] | 2016 | Multiple | Interviewees (n=28531) | Observational | 0.70 | Multiple |
| Kaur [83] | 2016 | India | Teachers (n=253), Children (n=30298) | Observational | 0.35 | Orbis International and Indian Council of Medical Research |
| Khair [52] | 2020 | Bangladesh | Individuals (n=299) | RCT | 0.85 | Nil |
| Kilangalanga [41] | 2019 | Democratic Republic of Congo | Children (n=11106) | Observational | 0.56 | University of Rostock and the Christoffel-Blindenmission |
| Krishnendu [49] | 2019 | India | Parents, school principals, medical officer, junior health inspector, Accredited Social Health Activists (n=not stated) | Qualitative | 0.60 | Nil |
| Latorre-Arteaga [20] | 2016 | Peru | Children (n=1909), Teachers (n=380) | Observational | 0.54 | Multiple |
| Loum [80] | 2017 | Uganda | Communities (n=2) | Observational | 0.75 | Task Force for Global Health and the National Institute of Allergy and Infectious Diseases |
| Lynch [53] | 2018 | Malawi | Children (n=30), community workers (n=14) | Mixed-methods | 0.77 | Multiple |
| Maitra [30] | 2021^ | India | Parents (n=30) | Qualitative | 0.65 | Not stated |
| Meredith [71] | 2012 | Multiple | Not stated | Observational | 0.45 | Sightsavers |
| Morjaria [88] | 2020 | India | Children (n=701) | Interventional | 0.86 | Multiple |
| Mousa [31] | 2015 | Egypt | Individuals (pre: n=525, post n=518) | Interventional | 0.43 | Nil |
| Mwangi [45] | 2020 | Kenya | Individuals (n=104) | Feasibility | 0.83 | The Queen Elizabeth Diamond Jubilee Trust |
| Mwangi [46] | 2020 | Kenya | Individuals (n=734) | Interventional | 0.68 | The Queen Elizabeth Diamond Jubilee Trust |
| Nicholls [70] | 2018 | Colombia | Individuals (n= 46 - 187 over 4 time periods) | Observational | 0.78 | Multiple |
| Nji [63] | 2021 | Cameroon | Community members (n=45), CDDs (n=26) | Qualitative | 0.75 | COUNTDOWN research consortium |
| Oyo-Ita [56] | 2021 | Nigeria | Children (n=2598) | Interventional | 0.81 | International Initiative for Impact Evaluation |
| Paudel [55] | 2021^ | Vietnam | Adults (n=400) | Interventional | 0.85 | Department of Foreign Affairs and Trade, Australian Government |
| Pehere [58] | 2021 | India | Community members (n= not stated) | Observational | 0.40 | Nil |
| Pradhan [54] | 2012 | India | Pregnant women and children (n=24046) | Observational | 0.39 | Not stated |
| Ramagiri [47] | 2020 | India | Individuals (n=267) | Mixed-methods | 0.59 | The Queen Elizabeth Diamond Jubilee Trust |
| Rasoloniaina [42] | 2019 | Madagascar | Children (n=4859) | Interventional | 0.55 | Seva Canada |
| Rono [81] | 2021 | Kenya | Individuals (n=128591) | Interventional | 0.88 | Wellcome Trust and The Queen Elizabeth Diamond Jubilee Trust |
| Sarma [57] | 2019 | Bangladesh | Key informants (n=58), caregivers (n=574), service providers (n=156), community members (n=28395077) | Mixed-methods | 0.80 | Gavi |
| Seelam [23] | 2021 | India | Key informants (n=19) | Qualitative | 0.80 | Multiple |
| Shija [76] | 2012 | Tanzania | Key informants (n=197), health workers (n=63), children (n=571) | Observational | 0.40 | Academy for Educational Development |
| Shukla [87] | 2020 | India | Accredited Social Health Activists (n=102) | Interventional | 0.73 | Sightsavers |
| Tadesse [50] | 2017 | Ethiopia | Children (n=1358) | Interventional | 0.82 | Addis Ababa University |
| Tao [43] | 2019 | China | Patients (n=470) | Interventional | 0.71 | Not stated |
| Tataryn [77] | 2017 | Malawi | Children (n=338200) | Observational | 0.77 | Multiple |
| Teerawattananon [85] | 2014 | Thailand | Students (n=5885) | Mixed-methods | 0.74 | National Health Security Office |
| Thummalapalli [24] | 2013 | India | Teachers (n=104), children (n=11452) | Interventional | 0.78 | Yale University |
| Tidwell [25] | 2019 | Kenya | Children (n=1340) | Interventional | 0.82 | The Queen Elizabeth Diamond Jubilee Trust |
| Weldegebreal [64] | 2014 | Ethiopia | Community members (n=418) | Observational | 0.86 | Addis Ababa University |
| York [32] | 2015 | Tanzania | Community members (n=482 surveys, n=42 FGD), CHWs (n=3), CDDs (n=5) | Mixed-methods | 0.65 | Sigma Theta Tau International |
