# Supplementary material for: Integrated people-centered eye care: A scoping review on engaging communities in eye care in low- and middle-income settings
Source: PLoS One. 2023 Jan 19;18(1):e0278969. doi: 10.1371/journal.pone.0278969 (PMC9851534; doi:10.1371/journal.pone.0278969)
Supplement: Attachment — Submitted filename: Response to reviewers.docx [file pone.0278969.s004.docx]

November 16^th^, 2022

Dear PLoS One Editors,

**Re: PONE-D-22-22189 Integrated people-centered eye care: A scoping review on engaging communities in eye care in low- and middle-income settings**

Thank you and reviewers for feedback on the above manuscript. Please find below our responses to Reviewer 1’s comments and journal requirements.

We look forward to your response.

Yours sincerely,

Dr Ling Lee
*PhD, BOptom(Hons), PGCertOcThera*

**Response to Reviewer**

**Reviewer 1:**

***On Line 249: Did the author mean 'lacking promotion of onchocerciasis prevention and control beyond the life of the project"?***

Response: Thank you for identifying our error. We have revised to include ‘prevention and control’ in the manuscript.

***Line 267 to 268: Were the reasons for male distributors less likely to access female due to religious or cultural beliefs?***

Response: Male distributors were less likely to access females due to cultural and health seeking behavioural norms. Additional details have been included.

**Journal Requirements**

***Please review your reference list to ensure that it is complete and correct. If you have cited papers that have been retracted, please include the rationale for doing so in the manuscript text, or remove these references and replace them with relevant current references. Any changes to the reference list should be mentioned in the rebuttal letter that accompanies your revised manuscript. If you need to cite a retracted article, indicate the article’s retracted status in the References list and also include a citation and full reference for the retraction notice.***

Response: No retracted papers have been cited in the manuscript. Where required for consistency, journal abbreviations, doi links have been edited. Please get in contact if further amendments are required.
